# Supplementary material for: Shared Common Ancestry of Rodent Alphacoronaviruses Sampled Globally
Source: Viruses. 2019 Jan 30;11(2):125. doi: 10.3390/v11020125 (PMC6409636; doi:10.3390/v11020125)
Supplement: Supplementary file 1 [file viruses-11-00125-s001.pdf]

Table S1. List of all the reference sequences used for the ORF1b, S and N phylogenetic analyses along with their accession number.

Accession number	Virus name
**KY370050**	RtRl-CoV
**KF294380**	Lucheng Rn rat coronavirus
**KY370045**	RtMruf-CoV
**NC034972**	AcCoV-JC34
**DQ286389**	Feline coronavirus
**DQ811788**	TGEV Purdue P115
**NC030292**	Ferret coronavirus
**NC028824**	BtRf-AlphaCoV
**NC009988**	Bat coronavirus HKU2
**MF094688**	Swine acute diarrhea syndrome related coronavirus
**MG557844**	Swine acute diarrhea syndrome coronavirus
**KF530114**	Human coronavirus NL63
**KF514433**	Human coronavirus 229E
**KP890336**	Porcine epidemic diarrhea virus
**NC010438**	Bat CoV HKU8
**NC010437**	Bat coronavirus 1A
**EU420137**	Bat coronavirus 1B
**KY967735**	Wencheng Sm shrew coronavirus
**KT861628**	MERS coronavirus
**HM211101**	Bat coronavirus HKU9
**NC004718**	SARS coronavirus
**NC006577**	Human coronavirus HKU1
**NC012936**	Rat coronavirus Parker
**AC000192**	MHV
**NC017083**	Rabbit coronavirus HKU14
**KJ958219**	Human coronavirus OC43
**KF906249**	Dromedary camel coronavirus HKU23
**NC003045**	BCoV
**NC001451**	Avian infectious bronchitis virus
**KM888168**	RCoV/LEO/Rat sik/CHN/2007
**KM888163**	RCoV/RMU10 3212/Myo gla/GER/2010
**KU182955**	Bat coronavirus isolate YDB5C
**JQ410000**	Alpaca respiratory coronavirus
**KT368901**	Camel alphacoronavirus
**HQ728486**	Chaerephon bat coronavirus/Kenya/KY22/2006
**KY370043**	Rodent coronavirus isolate RtRn-CoV/YN2013
**KY370048**	Rodent coronavirus isolate RtMm-CoV/GD2015
**JX993987**	Bat coronavirus Rp/Shaanxi2011
**KY370046**	Rodent coronavirus isolate RtMruf-CoV-2/JL2014
**KY370047**	Rodent_coronavirus_isolate_RtAp-CoV/Tibet2014
**KY370051**	Rodent_coronavirus_isolate_RtBi-CoV/FJ2015
**KY370053**	Shrew-CoV/Tibet2014
**NC030886**	Rousettus_bat_CoV_GCCDC1
**KY799179**	Myotis_lucifugus_CoV
**KY370049**	RtNn-CoV/SAX2015
**KC545386**	ErinaceusCoV/2012-216/GER/2012
**HQ728483**	Rousettus_bat_coronavirus/Kenya/KY06/2006
**HQ728485**	Miniopterus_bat_coronavirus/Kenya/KY33/2006
**HQ728482**	Eidolon_bat_coronavirus/Kenya/KY24/2006
**HQ728480**	Cardioderma_bat_coronavirus/Kenya/KY43/2006
**JQ989273**	Hipposideros_bat_coronavirus_HKU10
**KF294357**	Longquan_Aa_mouse_coronavirus
**KM349744**	Betacoronavirus_HKU24

**KY370044**	RtAs-CoV/IM2014
**NC014470**	Bat_coronavirus_BM48-31/BGR/2008
**KT444582**	SARS-like_coronavirus_WIV16
**KF294371**	Longquan_Rl_rat_coronavirus
**MF167434**	Porcine_enteric_alphacoronavirus_GDS04
**KY370043**	RtRn-CoV/YN2013
**KY370048**	RtMm-CoV/GD2015
**JX993987**	Bat_coronavirus_Rp/Shaanxi2011
**KY370046**	RtMruf-CoV-2/JL2014
**KY370047**	RtAp-CoV/Tibet2014
**KY370051**	RtBi-CoV/FJ2015
**LC190906**	Canine_coronavirus
**KC522061**	Tylonycteris_bat_coronavirus_HKU4
**KC522091**	Pipistrellus_bat_coronavirus_HKU5
**EF434381**	Bat_coronavirus_Shandong/977/2006
**KJ473818**	BtRf-BetaCoV/HuB2013
**KU343208**	Bat_coronavirus_MsBtCoV/4068
**KT346372**	Scotophilus_kuhlii_coronavirus
**DQ666343**	Bat_coronavirus_HKU7

Table S2. List of all the reference sequences used for the Cytochrome b phylogenetic analysis along with their accession number.

Accession number	Species
**NC001913**	*Oryctolagus cuniculus*
**NC035621**	*Rattus baluensis*
**EU273710**	*Rattus exulans*
**JQ823538**	*Rattus andamanensis*
**GU570660**	*Rattus leucopus*
**KU200226**	*Rattus_nitidus*
**NC023347**	*Rattus niobe*
**KT024821**	*Rattus norvegicus*
**JQ823537**	*Rattus rattus*
**JQ823541**	*Rattus tanezumi*
**AY389020**	*Apodemus latronum*
**AY389019**	*Apodemus ilex*
**AY389021**	*Apodemus uralensis*
**HM034867**	*Apodemus chejuensis*
**AY389003**	*Apodemus_peninsulae*
**AY389012**	*Apodemus_agrarius*
**AF159392**	*Apodemus flavicollis*
**AF159395**	*Apodemus sylvaticus*
**AY389010**	*Apodemus draco*
**AY389016**	*Apodemus chevrieri*
**AY513792**	*Microtus daghestanicus*
**AY513814**	*Microtus majori*
**AF163890**	*Microtus abbreviatus*
**AY513839**	*Microtus tatricus*
**AY513813**	*Microtus lusitanicus*
**AY513844**	*Microtus thomasi*
**KX581049**	*Microtus obscurus*
**KU214697**	*Microtus limnophilus*
**AY513797**	*Microtus duodecimcostatus*
**AF163891**	*Microtus californicus*
**AF163899**	*Microtus miurus*
**AY513823**	*Microtus rossiaemeridionalis*
**AF163898**	*Microtus middendorffi*
**AF159403**	*Microtus arvalis*
**AF159402**	*Microtus agrestis*
**AY513789**	*Microtus cabrerae*
**AF187230**	*Microtus longicaudus*
**KJ081954**	*Microtus fortis*
**KJ789597**	*Myodes rutilus*
**KR059903**	*Myodes rufocanus*
**FJ881480**	*Myodes glareolus*
**FJ234423**	*Myodes gapperi*
**KJ556708**	*Myodes andersoni*
**NC016427**	*Myodes regulus*
